# Salvage of an Incomplete Sandwich With a Covered Celiac Trunk and a “Floating” Superior Mesenteric Artery Stent in a Thoracoabdominal Aortic Aneurysm

**DOI:** 10.1177/15266028221090448

**Published:** 2022-04-13

**Authors:** Ryan Gouveia e Melo, Benedict Ginthoer, Carlota Fernández Prendes, Jan Stana, Konstantinos Stavroulakis, Barbara Rantner, Nikolaos Tsilimparis

**Affiliations:** 1 Department of Vascular Surgery, Ludwig Maximilian University Hospital, Munich, Germany

**Keywords:** failed endovascular, re-intervention, fenestrated, branched, F/B-EVAR, parallel grafts, sandwich, chimney, snorkel

## Abstract

**Purpose:**

To report a case of a patient with a large thoracoabdominal aortic aneurysm (TAAA) extent V treated with a custom-made fenestrated and branched endovascular repair (F/B-EVAR) after a failed and incomplete attempt of a Sandwich repair technique.

**Report:**

An 83-year-old patient was referred to our department after a failed attempt at endovascular repair of type V TAAA with a sandwich technique. The celiac trunk was inadvertently covered with the first endograft and a covered long superior mesenteric artery stent was placed and left facing upward inside the aorta. We performed a staged repair, by first catheterizing and stenting the celiac trunk and bringing it under and inside the main aortic endograft. In interval, a F/B-EVAR was performed using a bimodular custom-made device (CMD) with a proximal 2 branch module for the celiac trunk and superior mesenteric artery and distal module with fenestrations for both renal arteries. The intervention was successful, and the follow-up was uneventful at 6 months.

**Conclusions:**

Re-intervention after failed endovascular attempts of TAAA repair are technically challenging and require advanced endovascular techniques. The ability to construct CMDs allowed to extend repair to our patient which had severe anatomical constraints for other techniques.

## Introduction

Endovascular aortic repair of thoracoabdominal aortic aneurysm (TAAA) is now the first-line option in most centers.^1,2^ Repair using fenestrated and/or branched custom-made devices (F/B-EVAR) has become the choice of treatment in these patients.^1,2^ However, manufacture time,^3^ cost, and technical complexity have led to the development of other available options such as parallel grafts with chimney, periscope, and sandwich techniques.^4^ Durability of endovascular repair is still a question of debate and re-interventions are common after primary repair, especially for non-custom-made devices or techniques.^1,2^ Furthermore, failure of the primary attempt at endovascular aneurysm repair may complicate or even compromise future interventions.^5,6^

We report a case of a patient with a large TAAA extent V treated with a custom-made F/B-EVAR after a failed attempt of Sandwich repair technique.

## Case Report

An 83-year-old female patient was referred to our department after an initial attempt at endovascular treatment of a TAAA extent V. The aneurysm had a maximum diameter of 79 mm and was initially operated 3 months before referral to our center. The initial plan in the referring Hospital was to perform a Sandwich technique with a Chimney for the celiac trunk (CT) and superior mesenteric artery (SMA). However, during the procedure, the first thoracic graft (Valiant Navion VNMF3731C173TE, Medronic® Inc, Santa Rosa, Calif, USA) was deployed lower than intended and covered the CT, the SMA was stented with a Gore^®^ Viabahn^®^ VBX 7 x 79 mm (W. L. Gore & Associates, Flagstaff, Arizona, USA) and left inside the main thoracic graft, without completing the sandwich technique. A second attempt was made at catheterizing the CT to complete the repair but was unsuccessful and the patient was referred to our center for evaluation.

**Figure 1.**
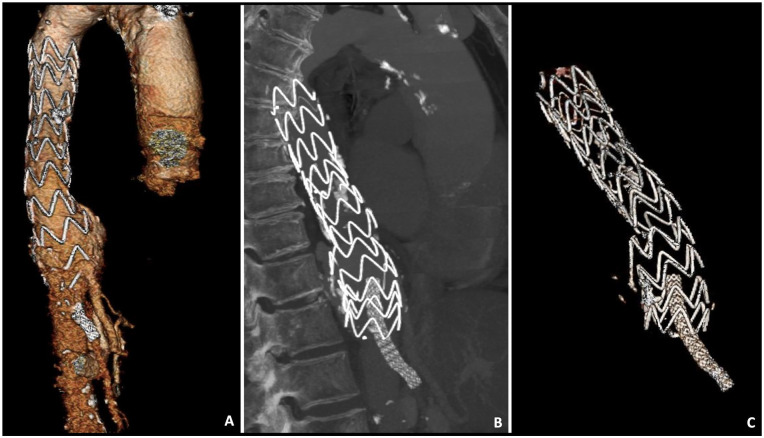
Preoperative celiac trunk-angiography: (A) 3D reconstruction, (B) sagittal view, and (C) 3D reconstruction showing the position of the stent grafts.

Diagnostic CT-angiography showed a patent but covered CT, a patent SMA with a long stent facing upward inside the thoracic aortic graft and a significant type Ib endoleak with no increase in size (Figure 1A–C). Other medical comorbidities included stage IV chronic kidney disease, arterial hypertension, cardiomegaly with signs of pulmonary hypertension, moderate aortic and mitral regurgitation, peripheral arterial occlusion disease, chronic obstructive pulmonary disease (COPD), and past-nicotine abuse.

The patient was operated in a staged procedure. The plan was to first stent the CT and bring the stent inside the main aortic graft, followed by a fenestrated and branched repair using a COOK® Zenith custom-made bimodular graft (COOK Medical^®^, Bloomington, Ind, USA) with a proximal part with 2 internal/external branches (for the CT at 1 o’clock and the SMA at 3 o’clock with a preloaded wire) and a distal part with 2 fenestrations for the renal arteries.

**Figure 2.**
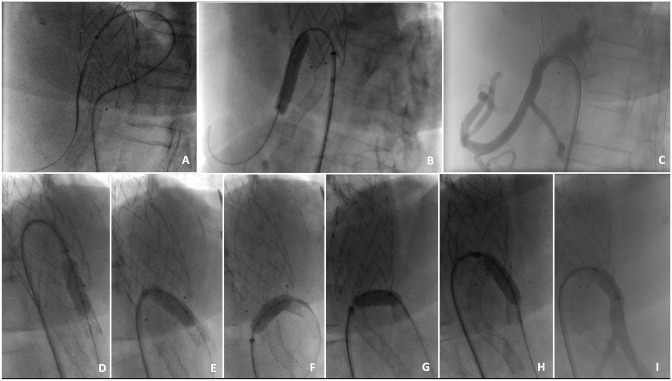
Intraoperative sequence of the first stage of the repair. (A, B, and C) Catheterization of the celiac trunk from outside the thoracic endograft and deployment of a covered stent. (D–I) Sequence showing the inflation of a balloon in the proximal two-thirds of covered stent followed by pulling and pushing maneuver to bring the stent inside the main aortic graft.

The first procedure was performed under local anesthesia and percutaneous femoral access. As the thoracic graft was not sealing distally, the aorta externally to the graft was catheterized, followed by catheterization of the celiac trunk using an UF^®^ catheter (Cordis^®^, Fremont, CA, USA) and a Terumo radiofocus® glidewire (Terumo^®^ Medical Corporation, Somerser, New Jersey). After careful advancement of the catheter into the common hepatic artery, the glidewire was exchanged with a stiff Amplatz^®^ wire (Boston Scientific^®^, Natick, MA) for support (Figure 2A). Following this a 7F COOK^®^ Flexor^®^ Ansel sheath was advanced inside the celiac trunk and a BeGraft Peripheral 8 x 38 mm (Bentley®, Hechingen, Germany) was deployed with half the stent inside the artery and half outside (Figure 2B and C). During de-inflation of the stent balloon, the sheath was re-advanced using the “swallowing” technique. The stent delivery system was removed and a 9 x 20 mm angioplasty balloon was then advanced into the proximal two-thirds of the stent graft, the sheath was pulled back just behind the balloon, and the balloon was inflated. After full inflation of the balloon, and supporting it on the sheath, both the balloon and the sheath were pulled back, thus bringing the stent down and performing an almost 180° bend making sure the proximal part of the stent was fully under the lower struts of the thoracic endograft. While keeping the balloon inflated, both the sheath and balloon were pushed upward and inside the thoracic graft. Using this maneuver, the stent was then brought from outside the graft to under and then inside the previously placed thoracic graft (Figure 2D–I). The stent was then ballooned again to confirm complete dilation without kinking/crushing. A control angiography showed correct placement and patency of both SMA and CT stents.

**Figure 3.**
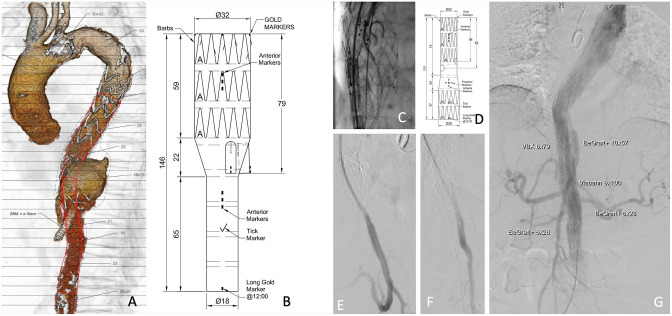
(A, B, and D) Custom-made graft plan and description. (C) Partial deployment of the branched graft and catheterization of the superior mesenteric artery. (E–G) Control angiographies. SMA, superior mesenteric artery.

After delivery of the custom-made endograft (Figure 3A, B, and D), the repair was completed under general anesthesia, bilateral percutaneous femoral access, and right cut-down axillary access. The procedure was performed in a fully equipped hybrid operating room with fusion imaging.

From right axillary access, a 12F sheath (COOK^®^ Flexor^®^ Ansel sheath) was placed above the CT and SMA stents. The CT stent and artery were catheterized with an UF catheter (Cordis^®^) and an Amplatz^®^ wire (Boston Scientific^®^) was placed inside to stabilize the stent. The first module of the custom-made device (CMD) graft (double branch; proximal diameter: 32 mm; distal diameter; 18 mm; length 146 mm) was then advanced and deployed partially until the branches were opened. The preloaded wire in the SMA branch was snared from above through the 12F sheath and a through-and-through access secured. Through the wire a 9F sheath (COOK^®^ Flexor^®^ Ansel sheath) was advanced to the branch and the SMA stent and artery were catheterized with an UF^®^ catheter and an Amplatz^®^ wire placed (Figure 3C). The graft was then fully deployed, followed by catheterization of the CT branch, stent and artery with a Bernstein^®^ catheter (Cordis^®^) and placement of an Amplatz^®^ wire with removal of the previously placed one. The CT was then bridged using a GORE^®^ Viabahn^®^ VBX Balloon Expandable Endoprosthesis 8 x 79 mm and the overlap inside the branch ballooned using a 9 x 20 mm balloon. The SMA was then bridged using a GORE^®^ Viabahn^®^ VBX 9 x 100 mm distally and a BeGraft plus 10 x 57 mm (Bentley^®^) proximally. Both control angiographies of the CT and SMA showed no signs of kinking, dissection, or endoleak (Figure 3E and F).

The second module of the CMD graft (2 fenestrations; proximal diameter: 22 mm; distal diameter 28 mm; length 131 mm) was then advanced inside the previous one, the fenestrations were aligned under fluoroscopy and fusion imaging and the graft deployed under constraining wires. The renal fenestrations and arteries were then catheterized, followed by placement of Rosen wires (COOK^®^ Medical), 6F sheaths, and parking of the bridging stents (Begraft peripheral 6 x 28 [Bentley^®^] on both sides). The graft was then fully deployed and both the thoracic and CMD modules overlap ballooned as well as the fenestrated module, using a 46 mm CODA^®^ balloon (COOK^®^ Medical). The sheaths were then pulled back, the renal stents deployed, and flaring was performed with a 9 x 20 mm balloon to seal and secure the fenestration bridging stents. As the infra-renal aorta was non-aneurysmatic, there was no need to extend the repair below the fenestrated module. A completion angiography showed patency of all target vessels with no endoleaks (Figure 3G). Both femoral access were closed with Proglides^®^ (Abbott^®^ Vascular Inc., Santa Clara, CA, USA) and the right axillary artery was closed with direct transverse suture.

**Figure 4.**
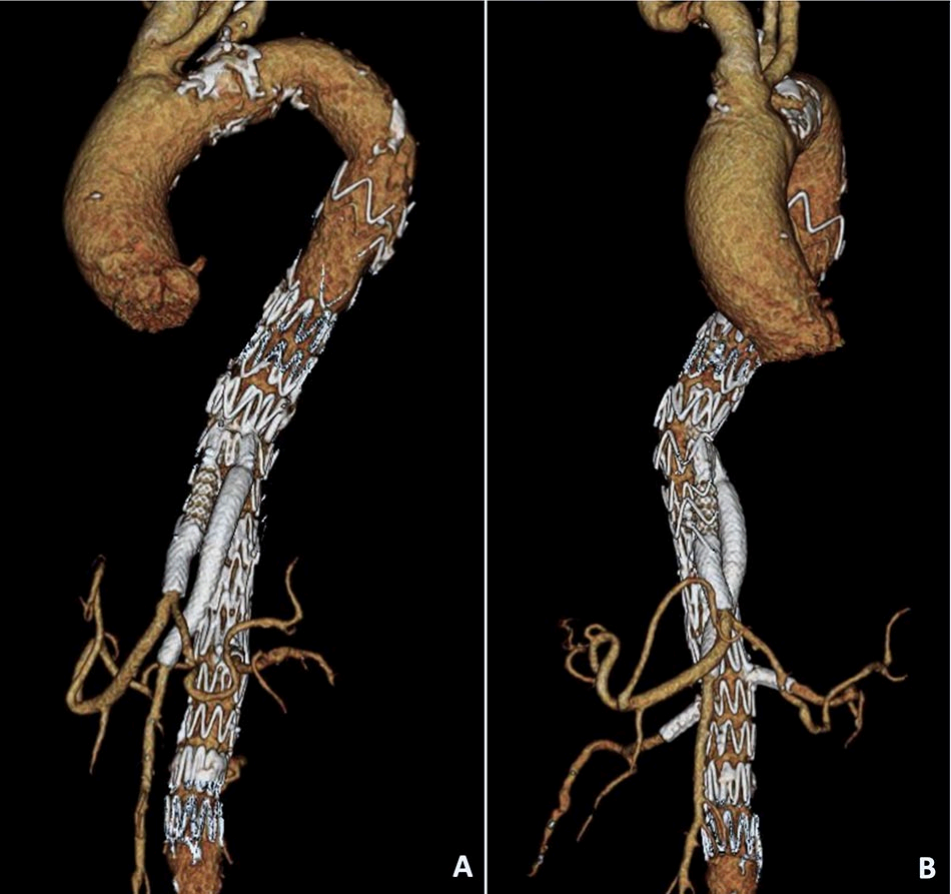
Postoperative 3D reconstruction of the celiac trunk-angiography.

Postoperative recovery was uneventful, and the patient was discharged 8 days after surgery. The first-month CT-angiography showed exclusion of the aneurysm, patency of all stents, and target vessels, with no signs of target vessel instability, endoleaks, or mural thrombus (Figure 4A and B). The patient is alive and well 3 months after surgery.

## Discussion

Our patient presented with a failed attempt of repair of a TAAA extent V with a Sandwich technique with a large type IB endoleak. The main difficulties were the fact that the CT was covered by the first thoracic aortic endograft and that the SMA was already stented with a long-covered stent placed inside the aorta. Several options at completing the repair were possible and were discussed.

Completion of the sandwich technique with a sole chimney for the SMA was a possibility; however, as the CT was patent, a type II endoleak from the CT was probable to keep the aneurysm perfused, in addition to the high risk of gutter endoleaks associated with parallel graft techniques, especially for TAAA aneurysms.^7^

Instead of trying to preserve the celiac artery, another option would be to occlude it with plugs/coils and perform the repair without including it. Although the celiac artery was already covered in our case, which could be an argument against including it in the repair, as the aneurysm had a significant type Ib endoleak and the celiac artery was patent on the CT-angiography, we could not be sure the patient would tolerate definite occlusion of the celiac and whether the observed patency was in fact from collateralization or antegrade flow from the endoleak. We believed that, if it was possible to incorporate the CT, would be optimal for both aneurysm sealing and to avoid complications such as hepatic ischemia. This is especially relevant, because other studies have shown that occluding the CT in TAAA endovascular repair is associated with higher morbidity and mortality.^8^

By deciding to perform the procedure in a staged manner, it allowed us to understand if we were able to bring the celiac artery stent inside the aortic endograft in order to incorporate it or not. Following this step, we could have completed the repair with a Sandwich technique, an off-the-shelf device, or a CMD.

As the aneurysm was large, we wanted to secure aneurysm sealing and so believed that the parallel graft technique was not the best option, because the risk of gutter endoleak is high.^7^

The option to use an off-the-shelf device, such as the t-branch (COOK^®^ Medical), was also complicated because there were already long-covered stents in SMA and CT. This meant the need to land the graft too far from the renal arteries (renal branches would end up 85 mm above the renal arteries) and compromise with bridging stent patency.^9^

Although the aneurysm had 79 mm, the patient was not symptomatic and there were no signs of rupture, so we opted for a CMD, which was requested with urgency.

Several options, however, were possible regarding the custom-made graft design. One possible easier option would have been to have a retrograde branch for the celiac artery and stent it from below, thus avoiding the extra maneuver of facing the celiac upward described above. We believed, however, this brought some disadvantages compared to the final selected design. First, by having an antegrade branch for the SMA and retrograde branch for the celiac would mean that bridging stents could cross each other, and risk being crushed or kinked. In addition, as the SMA stent was so high up from the renal arteries, a 2-model graft offered a way to overcome this. If a retrograde branch was used, this branch would probably be incorporated in the distal module and thus minimizing the possible overlap between both models, and for manufacturing purposes, it would be difficult to have a retrograde branch, as it would interfere with the renal fenestrations. In addition, fluid dynamics appears to be better in antegrade versus retrograde branches.^10^

The chosen custom-made design offered several advantages. As the top of the previously placed stents were at a long distance from the renal arteries, the bimodular nature of the device allowed to incorporate all target vessels, with branches and fenestrations suited to each target vessel. Furthermore, with this CMD, we were able to precisely choose the overlap between the previous thoracic endograft (3 complete stents) as well as proximal oversizing (32 mm CMD inside a 31 mm endograft). The fact that we were able to adjust distal diameter of the graft avoided the need to extend the repair to the aortic bifurcation, thus lowering the risk of spinal cord ischemia.^11^

## Conclusions

This case report illustrates the technical complexity of dealing with failed endovascular attempts at treating TAAAs. Advanced endovascular technical skills and the use of custom-made F/B-EVAR allowed for successful salvage of the aneurysm repair.
